# HEY1 promotes the development and metastasis of osteosarcoma through CD44/EGFR/FAK pathway

**DOI:** 10.1111/jcmm.70042

**Published:** 2025-06-18

**Authors:** Yuhang Liu, Hao Zhang, Xinzeyu Yi, Zheng Wang, Aixi Yu

**Affiliations:** ^1^ Department of trauma and microorthopaedics Zhongnan Hospital of Wuhan University Wuhan China

**Keywords:** Bioinformatic, CD44, focal adhesion pathway, HEY1, osteosarcoma

## Abstract

Osteosarcoma (OS) is a highly prevalent and deadly malignant tumour primarily affecting adolescents. However, the identification of new therapeutic targets remains an urgent need. The advent of bioinformatics technology has offered us a novel approach to screen key genes from diverse OS‐related databases, thereby providing valuable insights into the mechanistic understanding of OS prognosis. In this study, we comprehensively integrated multiple databases to identify the crucial oncogene, HEY1, which exerts a significant impact on OS prognosis. Subsequently, we conducted a experimental validations to explore influence of HEY1 knockdown on OS cells. HEY1 exhibited significant overexpression in OS tissues and cells and its silencing resulted in a significant inhibition of proliferation. The interaction between HEY1 and CD44 was identified through transcriptome sequencing and mass spectrometry analysis. Additionally, our findings suggested that HEY1 could potentially influence the EGFR‐FAK pathway. Further experiments established that HEY1 regulates the EGFR‐FAK pathway via CD44, thereby influencing the biological phenotype of OS cells. These findings were subsequently validated using in vivo animal models. In summary, HEY1 demonstrated significant overexpression in both OS tissues and cells, exerting a substantial impact on the prognosis of OS.

## 1 INTRODUCTION

Osteosarcoma (OS) is a malignant bone tumour primarily found in children and adolescents, but it can also occur in individuals over 50 years of age, particularly those with Paget's disease. It originates from primitive mesenchymal cells and commonly occurs in the long bones of the limbs, including the femur, tibia and humerus, in close proximity to the growth plate.^1^ Lung metastasis is a common and significant complication in patients with OS, frequently observed before the initiation of multi‐agent chemotherapy.^2^, ^3^ The survival rate of 5 years for patients is nearly 60%, and the prognosis is poorer for patients with metastatic or recurrent focus, with a survival rate of only 20%.^4^

Despite the absence of substantial improvements in survival rates over the past three decades, recent advancements in OS genetics and clinical treatment strategies suggest that we are on the cusp of a transformative era.^5^ Currently, standard treatments for adolescent OS patients encompass preoperative neoadjuvant MAP chemotherapy, which consists of methotrexate, doxorubicin and cisplatin, followed by postoperative adjuvant chemotherapy.^6^ In elderly patients, high‐dose methotrexate is often contraindicated due to its associated toxicity. Moreover, the utilization of doxorubicin or cisplatin is sometimes restricted by long‐term side effects. Instead, ifosfamide or etoposide are considered as potential alternatives.^7^ In patients with metastatic disease, the management approach is comparable to that of the primary tumour, involving neoadjuvant chemotherapy followed by surgical excision of all metastatic sites. The complete resection of all identifiable lesions has been associated with enhanced overall survival.^8^ In the 1970s, systemic chemotherapy was viewed with pessimism regarding its efficacy in treating solid tumours. However, recent years have witnessed remarkable advancements in the field of targeted therapies, prompting researchers to explore more precise and effective targets. This progress has revolutionized the treatment landscape for OS. Presently, we are experiencing a significant paradigm shift in OS management, driven by novel insights into OS genetics and the adoption of personalized treatment strategies tailored to individual genetic profiles.^9^ The genetic characteristics of OS present a highly intricate landscape compared to other human tumours. To effectively translate these genetic features into potential treatment targets, it becomes imperative to identify the specific subtype of OS based on its genetic profile and other relevant biological attributes, including the tumour's immune status. Such comprehensive understanding will aid in developing targeted therapeutic approaches.^3^

The advancement of targeted therapies, specifically tailored to known genetic mutations, has revolutionized cancer treatment. However, the application of this approach encounters challenges in OS due to the absence of prevalent activating kinase mutations, which are commonly observed in other malignancies.^10^ In contrast, OS exhibits a high prevalence of gene amplifications and mutations. The intricate mutation patterns found in OS pose challenges in extrapolating chemotherapy targets from data obtained in other tumour types. Notably, abnormal gene amplifications, including IGF1R, CCBE1, MYC, among others, have been identified in OS. Leveraging CRISPR‐Cas9 technology, these amplifications can be validated as potential drug targets^11^ . In a clinical trial evaluating regorafenib, a multi‐kinase inhibitor targeting VEGF, The regorafenib group demonstrated a median duration progression‐free survival of 3.6 months in patients with OS. In contrast, the placebo group had a median progression‐free survival of 1.7 months.^12^ Targeting the immune system directly has emerged as a promising approach in OS research, bypassing the complexities associated with the tumour's genetic landscape. A study demonstrated the favourable inhibitory effect on OS growth by combining anti‐GD2 and anti‐HER2 antibodies that target T cells. Furthermore, the addition of a PD‐L1 antibody further enhanced the therapeutic efficacy of this combination.^13^ The widespread utilization of immune checkpoint inhibitors has prompted the urgent need for effective methods to predict treatment efficacy and minimize side effects, representing a crucial research challenge in current immunotherapy.

The significant success of targeted therapies and immunotherapies, including novel biological agents, in treating OS highlights their potential for further therapeutic advancements. In order to optimize the effectiveness of OS treatment, it is crucial to acquire a comprehensive understanding of the molecular and biological mechanisms underlying its initiation and progression. This understanding will enable the identification of additional potential biomarkers and novel drug targets, fostering continuous improvements in OS treatment outcomes.

HEY1, a member of the HEY family of basic helix–loop–helix (bHLH) transcription factors, is implicated in the mechanism of OS prognosis. These transcription factors play a vital role in modulating the expression of target genes by selectively binding to specific DNA sequences, thereby exerting precise control over the strength, timing and spatial distribution of gene expression. The aberrant regulation of transcription factors is intricately linked to the initiation, progression and metastasis of tumours, including OS.^14^ HEY1, a transcription factor, has emerged as a significant player in driving tumour progression across diverse cancer types, including OS. Notably, In a study by Yihuan Pu et al., the upregulation of HEY1 was observed in melanoma cells, leading to enhanced invasion and metastasis by modulating the GRB2/PI3K/AKT signalling pathway.^15^ Recent research findings have indicated the overexpression of HEY1 in human OS cells, thereby promoting their invasiveness and facilitating lung metastasis through the upregulation of MMP9 expression.^16^ In the process of epithelial‐mesenchymal transition (EMT), which is regulated by the transforming growth factor‐β (TGF‐β), the crucial role of HEY1 expression has been uncovered by Jing Yang et al. EMT is a cellular plasticity mechanism frequently observed during the advanced stages of carcinogenesis.^17^

These findings underscore the substantial contribution of HEY1 to tumour development, highlighting its promising prospects as a novel target for therapeutic interventions. However, few studies have been performed on the specific molecular mechanisms of HEY1 in OS.

Our study employed bioinformatics techniques to perform a comprehensive analysis across multiple databases, revealing HEY1 as a significantly differentially expressed gene with a profound impact on the prognosis of OS patients. By delving into the intricate molecular pathways modulated by HEY1, we have deciphered the underlying mechanisms that drive proliferation, invasion and metastasis in OS cells. The results in our research underscore the promising role of targeting HEY1 as a therapeutic approach for treating OS. This significant discovery provides a solid theoretical basis for the diagnosis and management of this disease, paving the way for improved clinical interventions.

## 2 MATERIALS AND METHODS

### 2.1 OS cell lines and tissue microarrays

We obtained a collection of human OS cell, including U2‐OS, 143B, MG‐63, HOS,and the cell lines of human osteoblast—hFOB 1.19, from Wuhan Procell Biological Company. OS tissue microarray was purchased from Zhongke Guang Hua Intelligent Biotechnology Co., Ltd., No. L714901, containing 70 cases of OS and 1 case of normal bone tissue, with clinical information of OS patients. The MG‐63, HOS and 143B cell were cultured using MEM complete medium and U‐2 cells were maintained in McCoy's 5A complete medium during the culture process. The osteoblasts cells were cultured using DMEM complete medium with 0.3 mg/mL G418 solution at 34°C.

### 2.2 Bioinformatics analysis

The GSE33382, GSE14359, GSE12865, GSE42352 and GSE70414 data sets were retrieved from the OS gene expression data available on the GEO (Gene Expression Omnibus) website at https://www.ncbi.nlm.nih.gov/geo/. The expression sets were analysed utilizing R software (4.0.2). The ‘limma’ package was employed for differential gene analysis (screening parameters: adj_*P* Value_ <0.05 & |log_2_FC| >2), ensuring robust statistical significance. Next, the expression of differential genes was extracted and analysed the relation with the prognosis of OS patients using R2 website, an online datamining and discovery platform (https://hgserver1.amc.nl/cgi‐bin/r2/main.cgi?open_page=login). GEPIA serves as a vital tool for the scrutiny of a substantial dataset comprising 9736 tumour samples and 8587 normal samples, meticulously sourced from the TCGA and GTEx projects (http://gepia.cancer‐pku.cn/index.html). In the GEPIA website, the TCGA SARC (Soft Tissue Sarcoma) dataset was utilized to assess the differential expression of the HEY1 gene between tumour and normal tissues. Furthermore, the association between HEY1 expression and the survival time of sarcoma patients was investigated.

### 2.3 qRT‐PCR

The entire RNA was extracted using Trizol reagent following the provided guidelines. The OD value for the extracted RNA samples were determined and it was stored at −80°C for follow‐up experiments. The target gene's CT value was detected after reverse transcription of the total RNA. The CT values of the genes were compared with the endogenous control (GAPDH) to calculate their relative expression level, which was expressed in the form of 2‐^ΔΔCt^. The primer sequences used for PCR: HEY1 sense, 5’‐TGCTCCATTACCTGCTTCTCAAA‐3′, anti‐sense, 5’‐TGCTCCATTACCTGCTTC TCAAA‐3′. GAPDH sense, 5’‐GGAGTCCACTGGCGTCTTCA‐3′, anti‐sense, GTCATG AGTCCTTCCACGATACC‐3′.

### 2.4 Cell transfection

The plasmid and small interfering RNA were transfected using lipofectamine 3000. Cells that exhibited favourable growth conditions were selected, digested and evenly spread into six‐well plates with a cell density adjusted to 70%–90%. Prior to transfection, the treated cells were allowed to equilibrate in serum‐free medium. Subsequently, 300 μL of Opti‐MEM medium, 3 μg of small interfering RNA or plasmid, and 150 μL of lipofectamine 3000 reagent were added to sterilized, enzyme‐free EP tubes and the above‐matched system was added to the six‐well plate. Following a 6–8 h incubation period, the old medium was substituted with MEM medium and cells were further cultured for 24–48 h. The sequences of small interfering RNA: siRNA NC: 5’‐UUCUCCGAACGUGUC ACGU‐3’, siRNA #1: 5’‐CGCAUCUCAACAACUACGCUUTT‐3′, siRNA #2: 5’‐GCA GGAGGGAAAGGUUACUUUTT‐3’, siRNA#3:5’‐CCCAACUACAUCUUCCCAGAU TT‐3′.

### 2.5 Construction of lentiviral stably transfected cell lines

Cells exhibiting optimal logarithmic growth and a density of approximately 90% were selected for the experiment. Before transfection, the medium was refreshed, and then 1.5 mL of lentivirus solution was added, along with a transfection enhancer at a concentration of 5 μg/mL. Once the cell density reached 90%, we measured the differential expression efficiency of genes and proteins and confirmed stable transfection before proceeding with subsequent experiments. The sequences of sh‐RNAs: shHEY1‐Forward, 5’‐GCAGGAGG GAAAGGTTACTTT‐3′, shHEY1‐Reverse, 5’‐GCAGGAGGGAAAGGTTACTTT‐3′.

### 2.6 CCK‐8 cell counting assay

The harvested transfected cells were subjected to centrifugation, resuspended in complete medium and subsequently counted for cell quantification. The cell suspension was seeded into 96‐well plates at a same density. A total of five time points were set at 0, 24, 48, 72 and 120 h, with five replicates per group. At each designated time point, CCK‐8 solution was gently introduced to plates, followed by a 2‐h culture period. Afterward, the OD values of the cells were quantified at a wavelength of 450 nm.

### 2.7 Cell scratching experiments

Transfect the OS cells and wait until the cell density reaches 90% before proceeding with the subsequent scratch assay. Using a 200 μL yellow tip, a vertical and uniform scratch was made on the cell culture plate. The plates were then incubated in an incubator with fresh serum‐free medium. The scratches were photographed at 0 h, 24 h and 48 h, starting at the time point of the scratch treatment. The migration distance of the scratch was calculated using ImageJ software to determine the healing rate of the scratch.

### 2.8 Transwell cell invasion assay

Firstly, the transwell chamber was coated with matrigel. Subsequently, cells were diluted to the desired concentration and 300 μL of the medium containing cells was meticulously introduced to upper chamber. Meanwhile, 700 μL of normal complete medium was added to wells of plates. Subsequently, the cells that had successfully migrated were fixed using paraformaldehyde solution and were subjected to staining using 0.5% crystal violet solution.

### 2.9 Clone formation experiments

The transfected cells were centrifuged, resuspended. Cells were added to six‐well plate and cultured for about 3 weeks until obvious cell colonies developed. Cell colonies were fixed using paraformaldehyde and subsequently stained using 0.5% crystal violet and photographed.

### 2.10 Apoptosis detection experiment

The cells were digested (using EDTA‐free trypsin), centrifuged and the supernatant was removed. 100 μLof cell suspension was added in a special flow tube followed by 5 μL ANNEXIN V/FITC, which was incubated in dark for 5 min; Finally, 5 μL PI solution was added and 400 μL PBS solution to make up the volume, the assay was performed immediately.

### 2.11 EDU cell proliferation experiments

The transfected cells were placed on six‐well plates and cultured overnight. The working solution was prepared and cells were EDU‐labelled, fixed, washed and permeabilized. The permeabilized cells were washed with PBS and nuclei were subjected to staining using 1× HOECHST33342 staining solution, after incubation at room temperature, cells were rinsed with PBS, after which the cells were photographed.

### 2.12 Western blot

Protein was isolated using PMSF solution. And the concentration was accessed using BCA assay. Total protein and markers were electrophoresed, closed with a blocking solution for 2 h. This was followed by antibody incubation. After washing, ECL emitter was added and the target proteins were developed.

### 2.13 Transcriptome sequencing analysis

The total RNA was isolated to obtain eukaryotic mRNA, which was fragmented and synthesized into cDNA to reconstruct the transcriptional library; the raw sequencing data were uploaded to the machine. The data are then filtered and quality‐controlled, and new transcripts are predicted after comparison with the reference genome sequence; gene expression analysis and variable shear analysis are performed on the transcriptome library. The expression distribution of differential genes was subsequently analysed, including correlation analysis, clustering analysis, enrichment analysis, etc.

### 2.14 Immunoprecipitation (IP) and mass spectrometry

After spreading 143B cells into 10 cm cell culture dishes, transfect Flag‐HEY1 plasmid. The experimental group was treated with the addition of flag antibody, whereas the blank group was treated with an equivalent amount of IgG antibody. The gels were stained using a silver staining kit from Solebro and subsequently separated by grouping and the subsequent mass spectrometry analysis was performed by Shanghai Zhongke New Life.

### 2.15 Animal experiments

Before starting the experiment, the experimental protocol was submitted to the Ethics Committee of Zhongnan Hospital of Wuhan University for approval. 5 week‐old balb/c nude mouse were selected and placed in a sterile environment at the Animal Experimentation Centre. Five mice were used in each group based on the principle of ‘reduce, replace and optimize’. Sterilize the chest of nude mice and use a 1 mL syringe to aspirate the treated OS cell suspension and slowly inject it into the right axilla of nude mice under the skin. 100 μL per nude mouse; After 18 days, the diameter of subcutaneous tumours reached about 1 cm, and control rats showed advanced malignant tumour, the nude rats were anaesthetised and executed by breaking their necks and the subcutaneous tumours were dissected out and preserved using paraformaldehyde solution; the subcutaneous tumours were photographed according to the grouping, the volume was measured and recorded, the tissue was examined by immunohistochemistry. The caudal vein injection method was utilized to establish the lung metastasis mouse using 143B cells.

### 2.16 Data processing and statistical analysis

The results obtained from this experiment were analysed using R software (4.0.2) as well as GraphPad Prism. All experimental manipulations were performed in three and more biological replicates. Results were presented using mean ± SD. The statistical analysis was conducted using the Student's *t*‐test for experiments with two subgroups and one‐way analysis of variance (ANOVA) for experiments involving three or more groups. A significance level of *p* < 0.05 was employed to assess the statistical significance of differences between the two groups.

## 3 EXPERIMENTAL RESULTS

### 3.1 Expression of HEY1 in OS and its prognostic significance in OS patients

**FIGURE 1:**
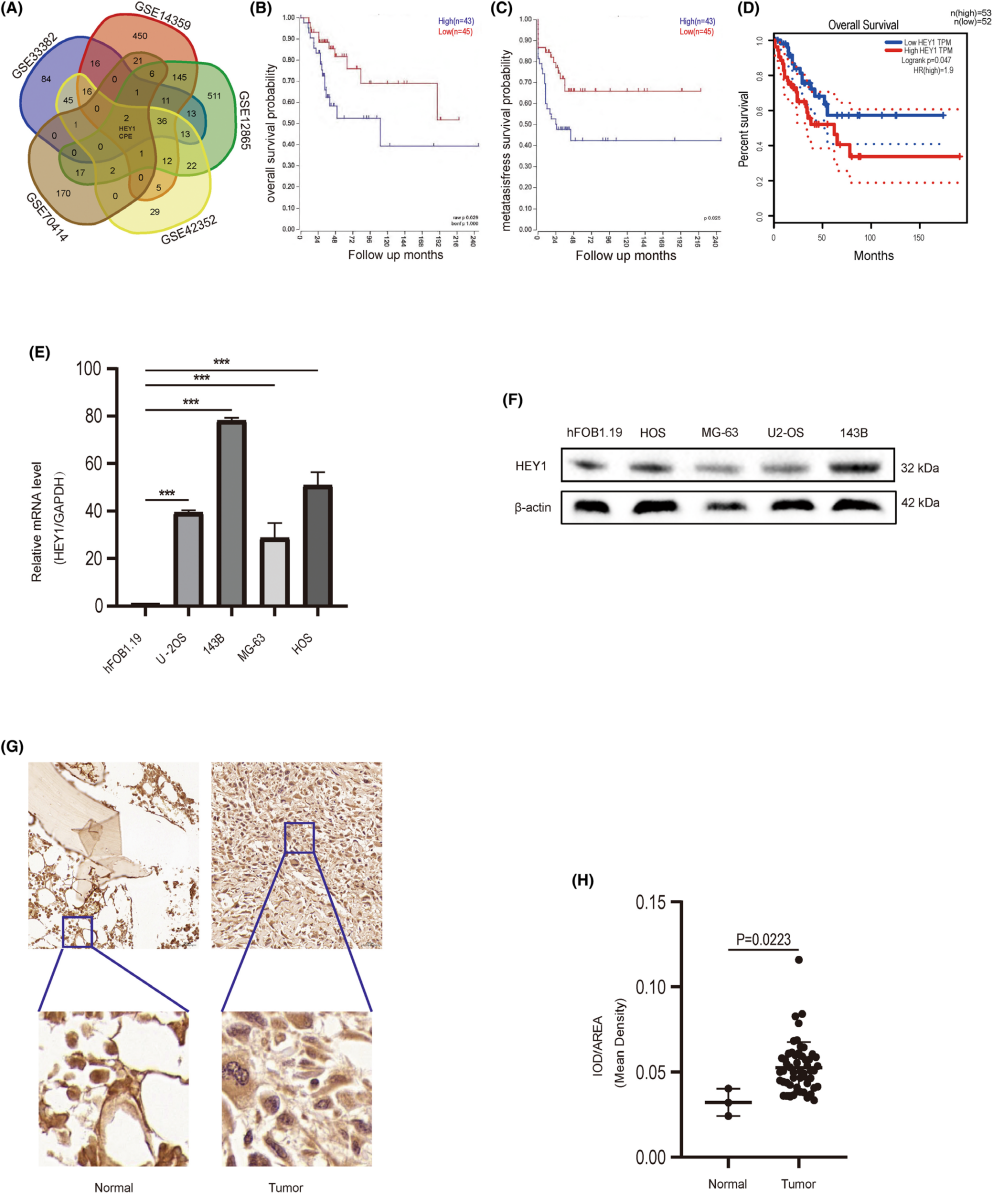
HEY1 exhibits significant upregulation in both osteosarcoma tissues and cells, correlating with patient prognosis. (A) Identification of commonly differentially expressed genes across five osteosarcoma databases. (B, C) Analysis of the association between HEY1 expression levels in the R2 database and overall survival as well as metastasis‐free survival in osteosarcoma patients. (D) Investigation of the relationship between HEY1 expression levels in the GEPIA database and overall survival in patients with soft tissue sarcoma. (E, F) Quantitative analysis of HEY1 expression using PCR and protein imprinting detection in osteosarcoma cells and normal osteoblasts. (G, H) Assessment of HEY1 expression levels in tumour tissues and normal tissues in osteosarcoma chips. *p < 0.05, **p < 0.01, ***p < 0.001.

To screen for differentially expressed genes that are significantly related to prognosis, we initially screened for genes exhibiting significant expression differences between OS tissues and normal tissues. We took the intersection of five GSE databases (GSE33382, GSE14359, GSE12865, GSE42352, GSE70414) according to the screening criteria (adj_*P*value_<0.05 &|log_2_FC| >2). Among the differentially expressed genes, HEY1 and CPE demonstrated remarkable consistency across all five datasets, underscoring their pivotal role in OS prognosis (Figure 1A). The results in R2 website demonstrated that HEY1 was significantly related to overall survival and metastasis‐free survival (*p* < 0.05) in OS patients. Furthermore, elevated levels of HEY1 expression correlate with a diminished overall survival period in individuals diagnosed with OS. (Figure 1B,C).However, there was no obvious association between CPE gene and prognosis of patients with OS (Data S1). Afterwards, validation within the GEPIA database further substantiated a notable association between the HEY1 gene and the prognostic outcomes in individuals diagnosed with soft tissue sarcoma. Furthermore, an elevated expression level of HEY1 consistently indicated an unfavourable prognosis in this patient cohort. Therefore, we selected HEY1 gene for the next step of our study. We conducted an analysis of HEY1 expression level across various OS databases.

Our findings demonstrate a statistically significant upregulation of HEY1 expression in tumour tissues (*p* < 0.01), which is consistent with our previous prognostic analysis, suggesting that HEY1 functions as a proto‐oncogene. To further validate our findings, we utilized common OS cell lines and hFOB 1.19 (human SV40 transfected osteoblasts). Significantly higher HEY1 expression levels were indicated in all OS cell lines (*p* < 0.01), with relatively higher levels demonstrated by 143B and HOS cell lines. As a result, these particular cell lines were selected for further analyses (Figure 1E). The Western blot findings corresponded to the results obtained from polymerase chain reaction analysis (Figure 1F).

Immunohistochemical staining of HEY1 was performed on OS microarrays, and the corresponding sections were analysed to determine the mean optical density. The findings revealed a significant high expression in the mean optical density of HEY1 in OS tissue compared to normal bone tissue (*p* < 0.05), suggesting the role of HEY1 in the pathogenesis and advancement of OS (Figure 1G,H).

### 3.2 In vitro functional effects of HEY1 on OS cell lines

**FIGURE 2:**
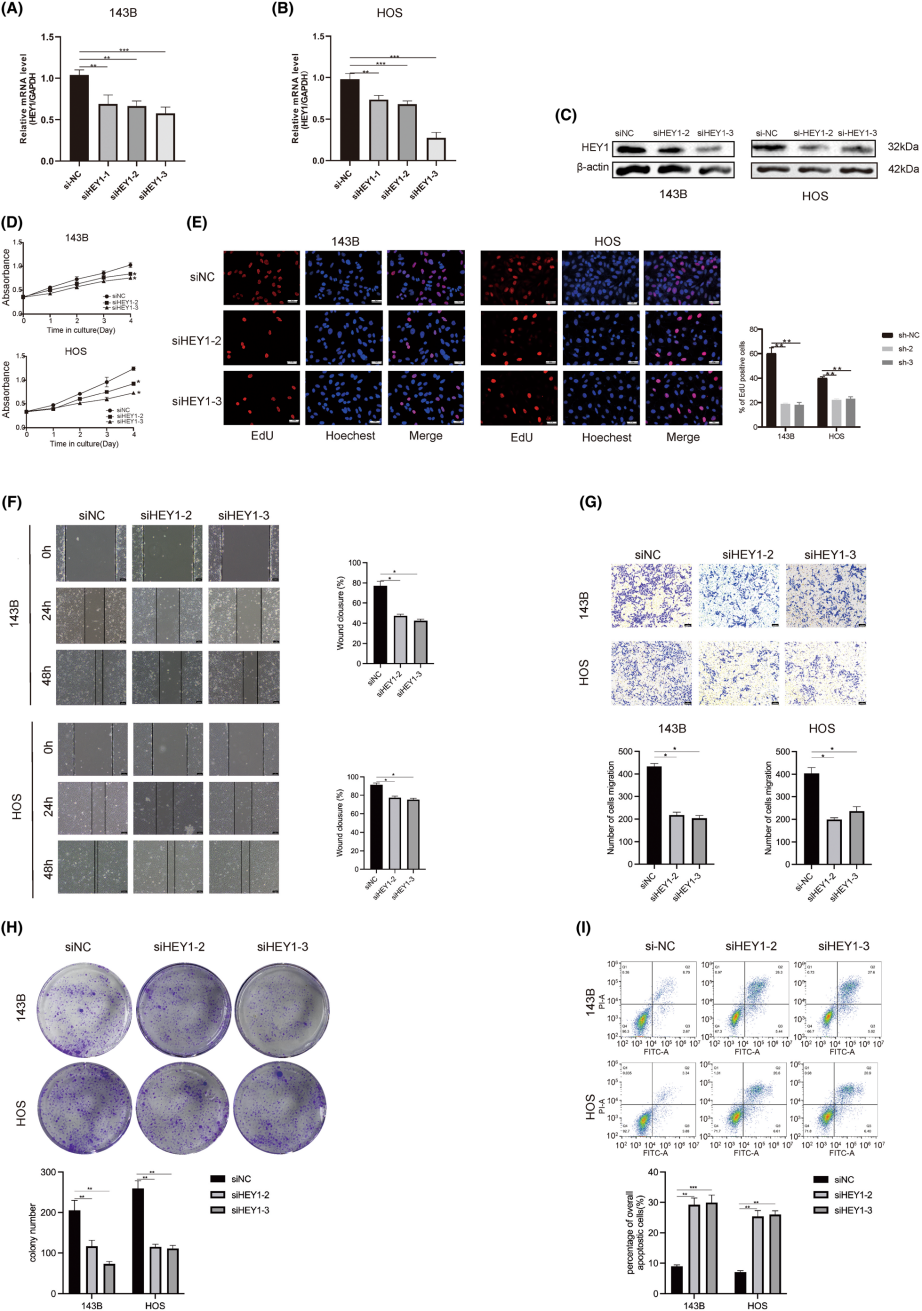
HEY1 plays a pivotal role in promoting the progression of osteosarcoma in vitro. (A–C) We assessed the levels of RNA and protein expression after suppressing HEY1. (D) Cell viability was evaluated using the CCK‐8 assay. (E) The EdU cell proliferation assay demonstrated the inhibitory effects of HEY1 knockdown on osteosarcoma cell proliferation. (F, G) Findings from the cell scratch assay and transwell assay indicate that HEY1 knockdown suppresses the migration and invasion of osteosarcoma cells. (H) The colony formation assay confirms the inhibitory effect of HEY1 knockdown on the formation of osteosarcoma cell colonies. (I) Flow cytometry apoptosis assay results highlight the promotion of osteosarcoma cell apoptosis achieved through HEY1 knockdown. *p < 0.05, **p < 0.01, ***p < 0.001.

To further substantiate the importance of HEY1 in OS, we employed transfection techniques to silence HEY1 expression in OS cell. The efficacy of the knockdown plasmids on the OS cell lines was evaluated on the level of mRNA. The findings revealed siHEY1‐2 and siHEY1‐3 exhibited a more pronounced inhibitory effect on HEY1 expression than siHEY1‐1 (Figure 2A–C).

Therefore, we selected sh‐2 and sh‐3 for subsequent experiments. After transfection, the CCK‐8 results demonstrated a significant reduction in cell absorbance values following HEY1 knockdown In comparison to the control group (Figure 2D). This indicates that siHEY1‐2 and siHEY1‐3 can effectively inhibit cell proliferation in 143B and HOS cells (*p* < 0.05). Moreover, EdU assays were performed on HEY1 knockdown cell lines, demonstrating a notable reduction in the proportion of EdU‐positive cells subsequent to HEY1 knockdown (Figure 2E). These findings suggest a notable reduction in the percentage of actively dividing cells in OS following the downregulation of HEY1.

Additionally, we explored the impact of HEY1 knockdown on the migratory and invasive abilities of OS cells. The scratch assay results demonstrated a significantly lower healing rate in the knockdown group for both 143B and HOS cells compared to the control group (siNC) after 48 h (Figure 2F). Subsequently, results of invasion assay indicated that after 48 h, the control group (siNC) exhibited a notably higher cell counts that traversed the membrane compared to the siHEY1‐2 and siHEY1‐3 groups (Figure 2G). These experimental results demonstrate that knocking down HEY1 leads to a significant decrease in the invasion ability of OS cells.

At day 14, the number of cell colonies in the control condition (siNC) exhibited a significantly higher count compared to the siHEY1‐2 and siHEY1‐3 conditions, both in 143B and HOS cells (Figure 2H). Flow cytometry analysis indicated that knocking down HEY1 can significantly promote apoptosis in the OS cell lines (Figure 2I).

### 3.3 Interaction between HEY1 and CD44 in OS cells

**FIGURE 3:**
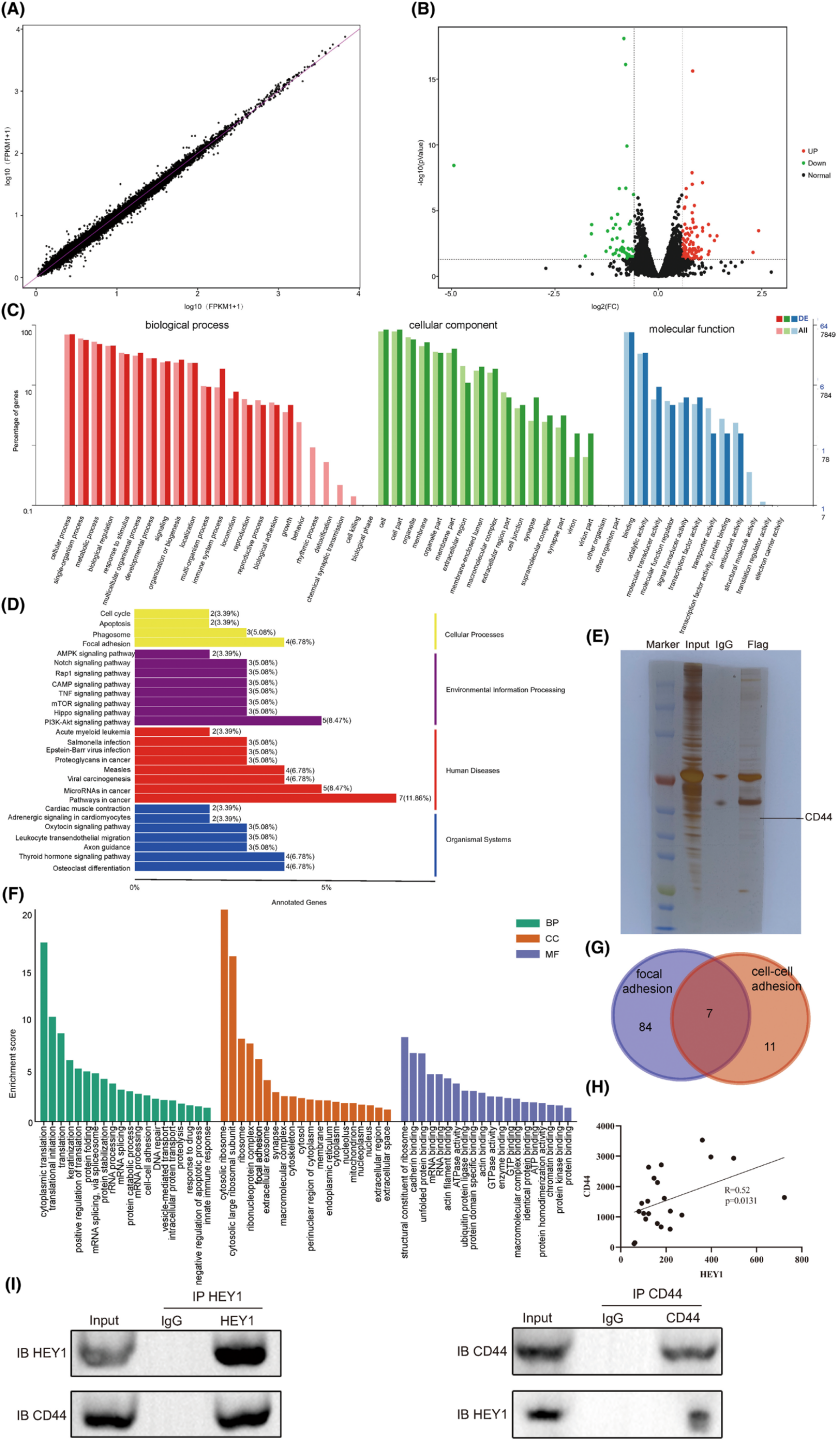
HEY1 exhibits an interaction with CD44. (A, B) Differential gene distribution and volcano plot obtained from high‐throughput transcriptome sequencing. (C, D) GO functional enrichment analysis and KEGG pathway enrichment analysis based on transcriptome sequencing. (E) Results of silver staining on immunoprecipitation gel. (F) GO functional enrichment analysis of proteins identified through protein mass spectrometry. (G) Shared proteins in the cell–cell adhesion and focal adhesion pathways. (H) Correlation analysis of HEY1 and CD44 expression levels in osteosarcoma databases. (I) CO‐IP results confirmed the interaction between HEY1 and CD44. *p < 0.05, **p < 0.01, ***p < 0.001.

To investigate HEY1's role in OS progression, we knocked down HEY1 in 143B cells and compared them to control cells transfected with empty plasmids. The expression correlation plot and volcano plot are shown in Figure 3A,B. Transcriptome sequencing analysis revealed enrichment of genes exhibiting differential expression in pathways associated with cell cycle regulation, apoptosis and focal adhesion. GO analysis indicated involvement in biological adhesion and cell junctions. The genes with altered expression levels were predominantly enriched in key pathways such as cell cycle regulation, apoptosis, focal adhesion and other related pathways, according to the KEGG pathway enrichment analysis. (Figure 3C,D).

To identify proteins that may interact with HEY1, we first transfected the HEY1 overexpression plasmid into 143B cells to evaluate the effect of HEY1 overexpression. We then used IP to isolate proteins that bind to HEY1 and performed silver staining on the gel (Figure 3E). The gel results were then subjected to mass spectrometry analysis. The results of gene ontology (GO) enrichment of the identified proteins demonstrated their enrichment in pathways such as cell–cell adhesion and focal adhesion (Figure 3F). Based on our comprehensive analysis, it can be inferred that HEY1 is likely to be involved in the focal adhesion pathway in OS, thus supporting our previous findings. Consequently, we obtained a list of seven proteins that are likely to interact with HEY1 and affect cell adhesion by overlapping the proteins associated with both the cell–cell adhesion and focal adhesion pathways (Figure 3G). Among them, we paid particular attention to CD44, a transmembrane glycoprotein which exerts a crucial role in cell–cell interaction and intracellular signalling pathways. CD44 can promote tumour cell proliferation, invasion, angiogenesis and adhesion when it binds to its ligand^18^. Furthermore, there are several activation pathways for CD44, including PI3K/AKT, Ras‐MAPK and EGFR/FAK pathways.^19^ The focal adhesion pathway plays a crucial role in regulating various cellular processes, such as proliferation, migration, invasion and apoptosis. focal adhesion kinase (FAK) is a critical component of this pathway^20^.

Based on our mass spectrometry and transcriptome results, we hypothesized that HEY1 activates the EGFR/FAK pathway by interacting with CD44. To test this hypothesis, we validated the correlation between HEY1 and CD44 using the OS database (GSE66673) (Figure 3H). Our results showed that the expression levels of HEY1 and CD44 were significantly correlated (Spearman correlation coefficient *R* = 0.52, *p* = 0.0131).

To further validate the interaction between HEY1 and CD44, we conducted co‐immunoprecipitation (CO‐IP) and immunoblotting assays in 143B cells. Our findings revealed that antibodies against both HEY1 and CD44 could co‐precipitate each other's antigens, indicating a potential interaction between HEY1 and CD44 within the 143B cell line (Figure 3I).

### 3.4 HEY1 modulates the biological processes of OS cells, including proliferation, migration and other relevant functions, by regulating CD44

**FIGURE 4:**
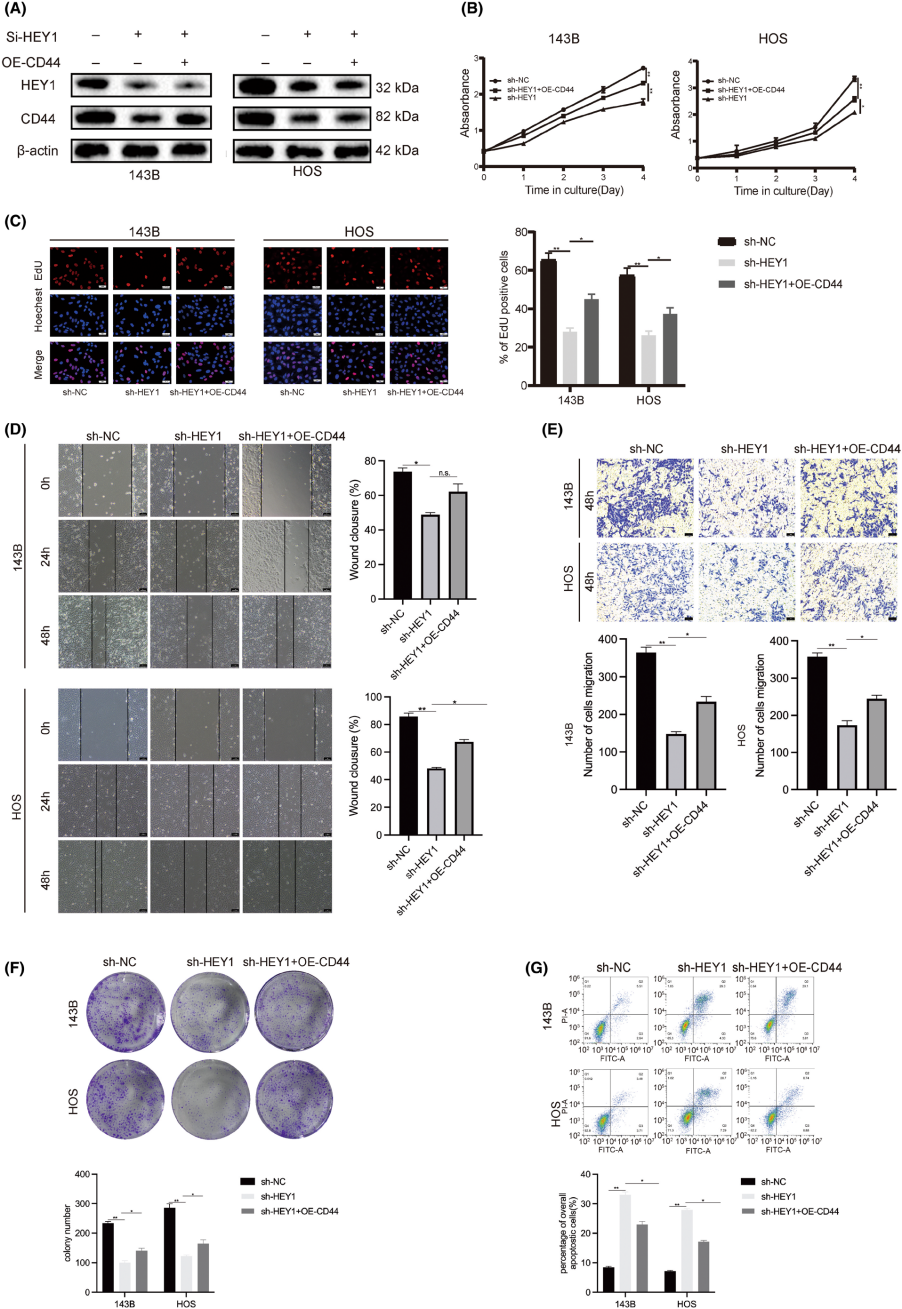
HEY1 promotes osteosarcoma cell invasion by activating CD44. (A) The protein level of CD44 was altered following HEY1 knockdown. (B, C) CD44 reversed the inhibitory effect of HEY1 knockdown on cell proliferation. (D, E) CD44 reversed the inhibitory effect of HEY1 knockdown on cell migration and invasion. (F) CD44 reversed the inhibitory effect of HEY1 knockdown on cell colony formation. (G) CD44 reversed the apoptosis‐promoting effect of HEY1 knockdown. *p < 0.05, **p < 0.01, ***p < 0.001.

To understand how HEY1 affects OS progression through CD44 regulation, we performed functional rescue experiments. The knockdown of HEY1 resulted in a notable reduction in the levels of CD44 protein in both the 143B and HOS cell lines. (Figure 4A). The ectopic expression of CD44 reversed the suppressive impact of HEY1 knockdown on the proliferation of OS cells, as demonstrated by CCK8 and EDU assays (Figure 4B,C).

The scratch and transwell invasion assays revealed CD44 can restore the migratory and invasive capabilities of OS cells following HEY1 gene knockdown. Plate colony formation assays revealed that co‐transfection of sh‐HEY1 and OE‐CD44 resulted in an intermediate number of cell colonies compared to the control and HEY1 knockdown‐only groups (Figure 4D,E). Flow cytometry analysis revealed a marked increase in the overall rate of apoptosis in the sh‐HEY1 + OE‐CD44 group compared to the group transfected with sh‐HEY1 alone. (Figure 4F,G).

### 3.5 Study on the mechanism and role of HEY1 in regulating OS through modulation of EGFR‐FAK pathway

**FIGURE 5:**
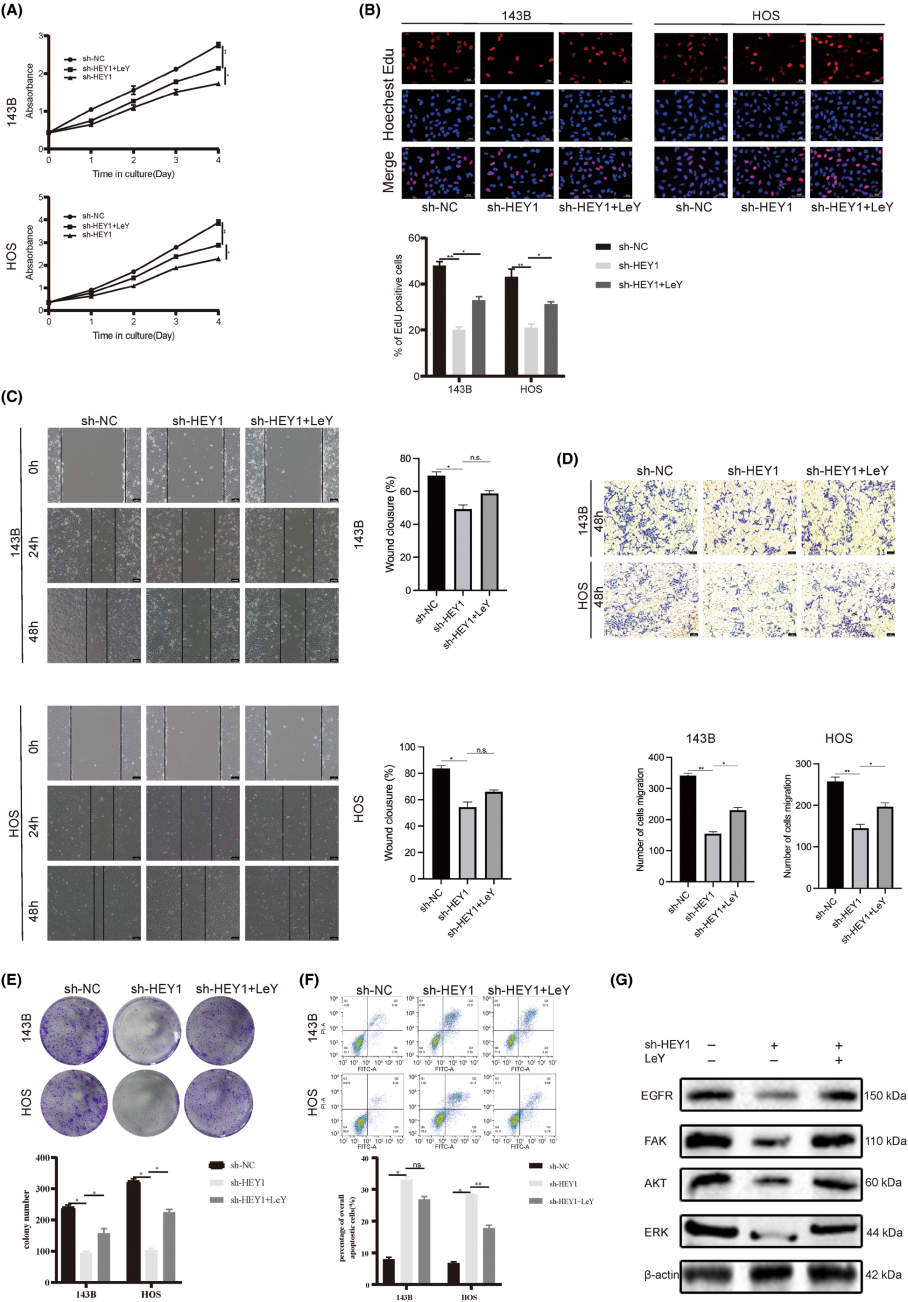
HEY1 promotes osteosarcoma progression through the CD44/EGFR/FAK pathway. (A, B) LeY reversed the inhibitory effect of HEY1 knockdown on osteosarcoma cell proliferation. (C, D) LeY reversed the inhibitory effect of HEY1 knockdown on osteosarcoma cell migration and invasion. (E) LeY reversed the inhibitory effect of HEY1 knockdown on osteosarcoma cell colony formation. F. LeY reversed the pro‐apoptotic effect of HEY1 knockdown. (G) Knockdown of HEY1 resulted in reduced expression levels of EGFR, FAK, Akt, and ERK, which were restored by LeY. *p < 0.05, **p < 0.01, ***p < 0.001.

In the previous section, we hypothesized that HEY1 could activate the EGFR‐FAK pathway through CD44, which we have shown can be regulated by HEY1. Therefore, we used the FAK activator Lewis Y tetrasaccharide (LeY) to restore the effect of silenced HEY1 on OS cells. In Figure 5A,B, the results from CCK‐8 and EDU assays revealed that LeY could counteract the suppressive impact of HEY1 downregulation on cell proliferation. The findings from cell scratch and transwell invasion assays demonstrated that the sh‐HEY1 group exhibited substantially reduced cell migration and invasion capabilities compared to the sh‐NC group. However, these effects were reversed when FAK was overexpressed, effectively restoring the impact of HEY1 silencing on cell migration (Figure 5C,D). The results obtained from the cell plate colony formation assay demonstrated that the presence of LeY effectively counteracted the inhibitory effect of sh‐HEY1 on colony formation (Figure 5E). Flow cytometry apoptosis assay results showed that LeY could restore OS cell apoptosis caused by silenced HEY1 (Figure 5F).

In order to validate the influence of HEY1 knockdown on the EGFR‐FAK pathway, we conducted Western blot analysis to evaluate the expression levels of proteins associated with the EGFR‐FAK pathway upon HEY1 silencing and supplementation of Lewis Y tetrasaccharide (LeY). As depicted in Figure 5G, the protein abundance of EGFR, FAK, AKT and ERK exhibited significant reductions upon HEY1 knockdown. Similarly to the functional recovery experiments discussed previously, The expression levels of FAK, AKT and ERK were upregulated to different extents following the addition of LeY, which provides further evidence of the regulatory role of HEY1 in the EGFR‐FAK pathway.

### 3.6 In Vivo Exploration of the impact of HEY1 on OS via the CD44/EGFR/FAK Pathway

**FIGURE 6:**
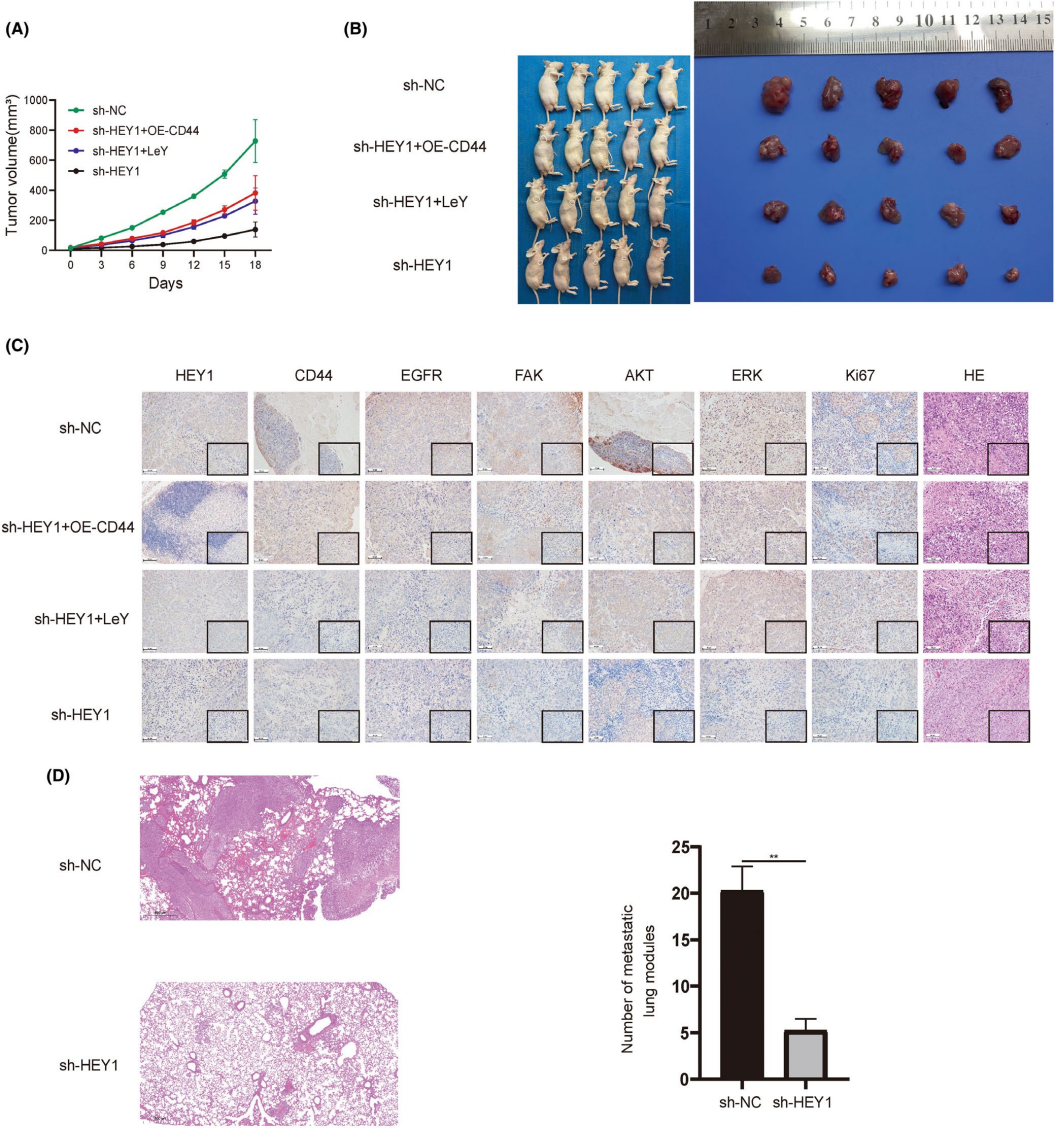
Knockdown of HEY1 suppresses the growth and metastasis of osteosarcoma in vivo. (A) Tumour growth curve. (B) Comparison of tumour volume after 18 days. (C) Immunohistochemical analysis and HE/Ki67 staining of subcutaneous tumours. (D) Knockdown of HEY1 inhibited pulmonary metastasis of osteosarcoma. *p < 0.05, **p < 0.01, ***p < 0.001.

In order to explore the in vivo impact of HEY1 on OS growth, stable knockdown of HEY1 and overexpression of CD44 were introduced in 143B cells. Subsequently, the tumour cells transduced with stable genetic modifications were employed to generate subcutaneous tumour models. The size of the subcutaneous tumours was monitored and recorded every 3 days to assess their growth kinetics. The findings revealed a significant reduction in the growth rate of the HEY1 knockdown group compared to the control group. Notably, the tumour volume in the group with CD44 overexpression or intratumoral LeY injection exhibited an intermediate level between these two groups. This suggests that knockdown of HEY1 significantly inhibited OS growth in vivo and that CD44 as well as LeY could reverse this inhibition (Figure 6A,B).

The levels of expression for HEY1, CD44, EGFR, FAK and ERK were significantly decreased in the sh‐HEY1 group compared to the control group, as observed through immunohistochemical analysis of the excised subcutaneous tumours. The downregulation of HEY1 was partially restored by overexpression of CD44 and addition of FAK activator LeY. This was further confirmed by the results of Ki67 and HE staining (Figure 6C). OS exhibits a substantially higher proclivity for pulmonary metastasis compared to other malignancies.

Based on these findings, our study aimed to explore the influence of HEY1 knockdown on the occurrence of pulmonary metastasis in OS. We intravenously injected 143B cells that had been stably transduced with sh‐HEY1 into nude mice, and then humanely euthanized the mice after they had developed mild cachexia. Lung tissues were subsequently subjected to HE staining. The results of our study demonstrated a significant reduction in both the size and quantity of lung metastatic nodules in the HEY1 knockdown group compared to the sh‐NC group (*p* < 0.05). Our results suggest that HEY1 knockdown can impede the metastatic propensity of OS (Figure 6D).

## 4 DISCUSSION

OS, a malignant tumour arising from bone or cartilage, primarily affects adolescents and middle‐aged individuals, with a higher incidence observed on the bones of the limbs. Existing treatment approaches for OS encompass surgical intervention, chemotherapy, radiation therapy, as well as targeted therapy. Despite these therapeutic options, challenges such as recurrence, metastasis and drug resistance persist. Therefore, continuous exploration of novel targets and the development of innovative treatment strategies and approaches are imperative.

The HEY1 gene, a member of the HES/HERP family, encodes a transcription factor.^21^ HEY1 can engage in interactions with other transcription factors, leading to the modulation of downstream gene expression, in addition to its direct regulation by the Notch signalling pathway.^22^ HEY1 has different functions in normal and tumour tissues, which may be related to its expression level, subcellular localization and interacting proteins^.^ ^23^ The HEY1 gene is associated with various tumours such as prostate cancer chondrosarcoma,^24^ and salivary gland adenoid cystic carcinoma.^25^ However, the mechanism of HEY1 in OS is still lacking detailed research. In this research, our primary objective was to examine the impact of HEY1 knockdown on OS cells.

Following the knockdown of HEY1, notable reductions were observed in the proliferation, migration and invasion capacities of OS cells. These findings strongly suggest that HEY1 functions as an oncogene in OS, corroborating earlier studies on the subject.^26^ Subsequently, we conducted CO‐IP and mass spectrometry analysis to identify CD44 as an interacting molecule with HEY1 in OS cells. CD44, a cell surface glycoprotein, has been implicated in oncogenesis across multiple cancer types and has been associated with the advancement and metastasis of OS.^27^ Therefore, we conducted functional recovery experiments by co‐transfecting sh‐HEY1 and OE‐CD44 to further validate the interaction between HEY1 and CD44, and their effects on the ability of proliferation, invasion of OS.

The findings from eukaryotic transcriptome sequencing and proteomics analysis revealed a potential association between HEY1 and the Focal adhesion pathway. Notably, previous research has demonstrated that CD44, acting as a cell surface receptor for receptor tyrosine kinases signalling, can modulate the Focal adhesion pathway through the regulation of EGFR.^28^, ^29^ The EGFR‐FAK pathway is a critical signalling pathway that encompasses the interactions between the epidermal growth factor receptor (EGFR) and FAK. This pathway holds significant importance in various cellular processes, including cell migration, proliferation, survival and EMT.^30^, ^31^ EGFR belongs to the tyrosine kinase receptor and can activate FAK by binding to its N‐terminal domain.^32^ Activation of the EGFR/FAK pathway can also regulate other signalling molecules, such as Src, Syk, ILK, etc. The EGFR/FAK pathway has been observed to exhibit abnormal expression or activation in numerous tumours and its dysregulation is closely linked to cancer invasion and metastasis^33^, ^34^.

The EGFR‐FAK pathway can participate in the signal transduction of OS proliferation and migration.^35^ Some findings have demonstrated the inhibitory effects of radioactive nanodrugs that specifically target EGFR on the growth and survival of OS cells. Further studies have demonstrated that circEMB, a circular RNA, facilitates the advancement and dissemination of OS by modulating the EGFR‐FAK pathway.^36^ Based on these research, it is postulated that HEY1 modulates the functionality of the EGFR/FAK pathway via CD44. In this study, we employed the FAK pathway activator LeY to assess its impact on the functional outcomes of HEY1 silencing in OS cells, including proliferation, migration, apoptosis and other cellular abilities. Our findings demonstrate that LeY administration reinstated the effects attenuated by HEY1 silencing, thus highlighting its potential in rescuing the altered cellular behaviours associated with OS. Finally, the subcutaneous tumour experiment results also showed that silencing HEY1 inhibits the growth of OS through the CD44/EGFR/FAK pathway. Furthermore, utilizing a mouse lung metastasis model, we observed a noteworthy suppression of OS lung metastasis upon HEY1 silencing. These findings provide additional evidence reinforcing the inhibitory effects of HEY1 gene silencing on the proliferative, migratory, invasive and metastatic capacities of OS cells. Moreover, this intervention was found to induce apoptosis in OS cells by modulating the CD44/EGFR/FAK pathway.

## 5 CONCLUSION

We employed bioinformatics analysis to screen and identify the HEY1 gene, which plays a vital role in the prognosis of patients with OS and exhibits over‐expression levels in both OS tissues and cells. Upon silencing HEY1, we observed its oncogenic nature, leading to a notable decrease in the proliferation, migration and invasion capabilities, accompanied by an increased rate of apoptosis. Subsequently, we used mass spectrometry, transcriptome sequencing and immunoprecipitation experiments to discover that HEY1 may affect OS progression through the CD44/EGFR/FAK pathway. To explore this discovery in more depth, we conducted additional experiments by introducing a lentivirus overexpressing CD44 and utilizing the FAK pathway activator LeY. These interventions were aimed at reinstating the suppressive effect of HEY1 silencing on the proliferation and invasion of OS cells. Subsequent analyses using subcutaneous tumour and lung metastasis models provided further evidence supporting the involvement of HEY1 in the progression of OS and the occurrence of distant metastasis. In conclusion, our findings strongly indicate that the suppression of HEY1 effectively hinders the development and metastasis of OS by attenuating CD44/EGFR/FAK pathway. These results highlight the potential of targeting the HEY1 gene as a novel therapeutic approach for treating OS.

## AUTHOR CONTRIBUTIONS

**Yuhang Liu:** Conceptualization (lead); data curation (lead); investigation (lead); writing – original draft (lead). **Hao Zhang:** Investigation (supporting). **Xinzeyu Yi:** Investigation (supporting). **Zheng Wang:** Investigation (supporting). **Aixi Yu:** Conceptualization (lead); project administration (lead).

## FUNDING INFORMATION

The present study was supported by the Medical Leading Talent Project of Hubei Province (grant. no. LJ20200405).

## CONFLICT OF INTEREST STATEMENT

The authors declare no competing interests.

## Supporting information


Data S1.

## Data Availability

The data used in this study are all from publicly available data.
