# Supplementary figures and images for: HEY1 promotes the development and metastasis of osteosarcoma through CD44/EGFR/FAK pathway
Source: J Cell Mol Med. 2025 Jun 18;29(12):e70042. doi: 10.1111/jcmm.70042 (PMC12175638; doi:10.1111/jcmm.70042)

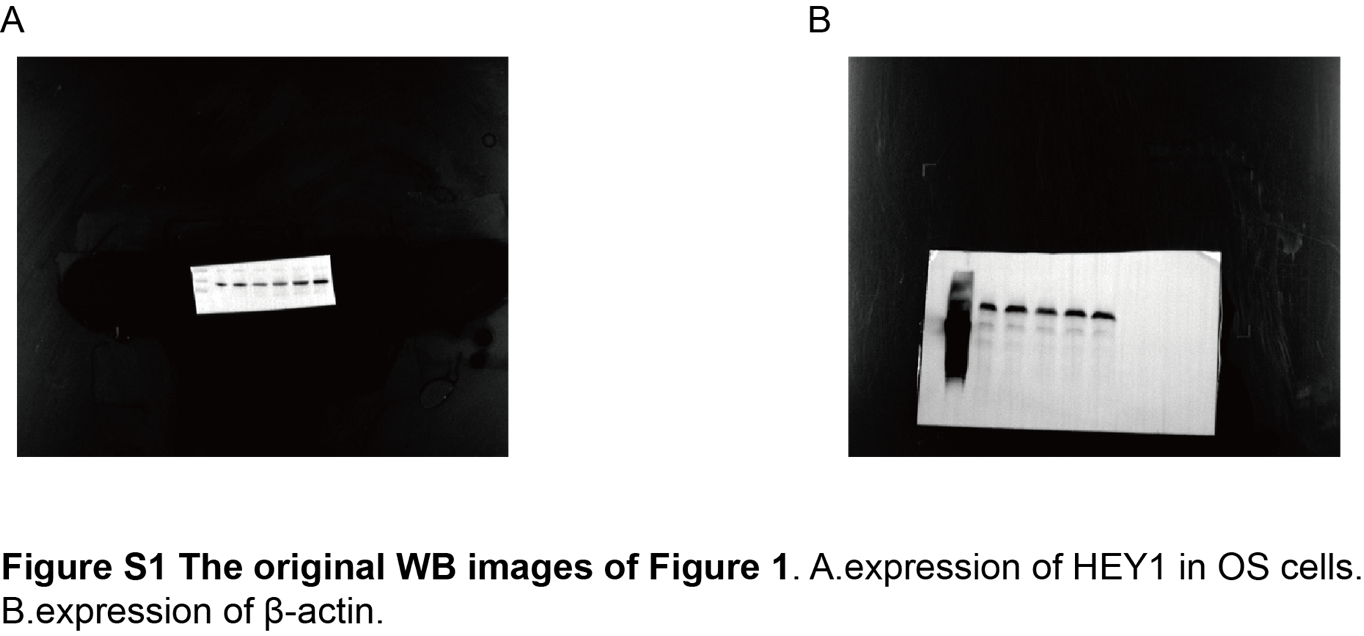


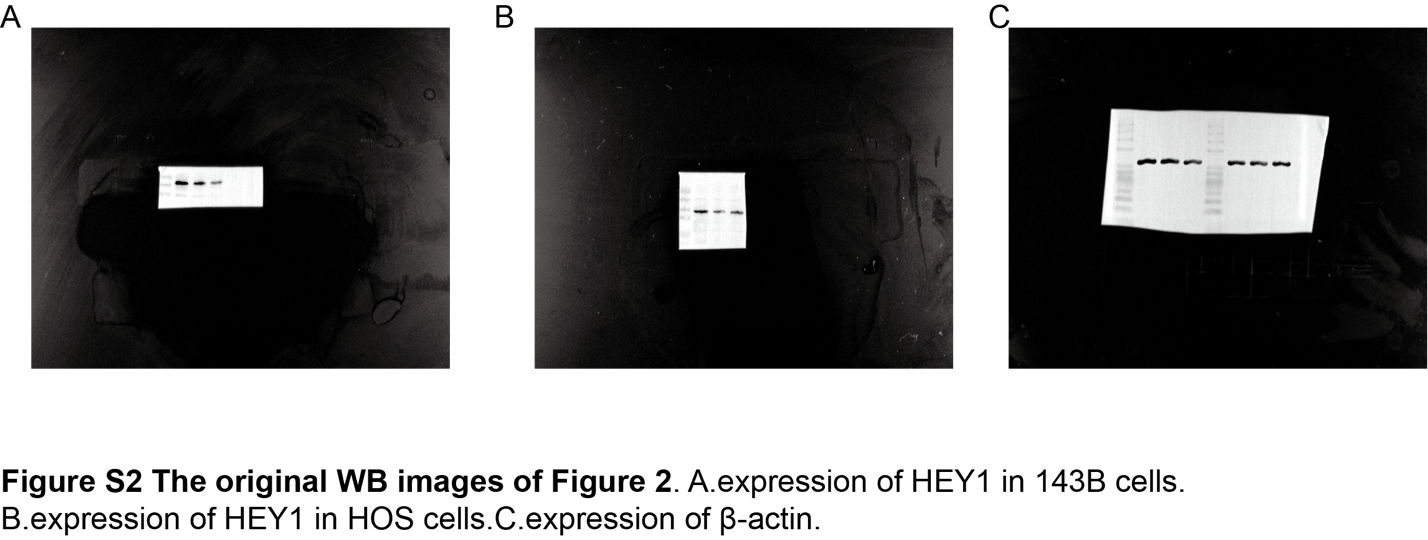


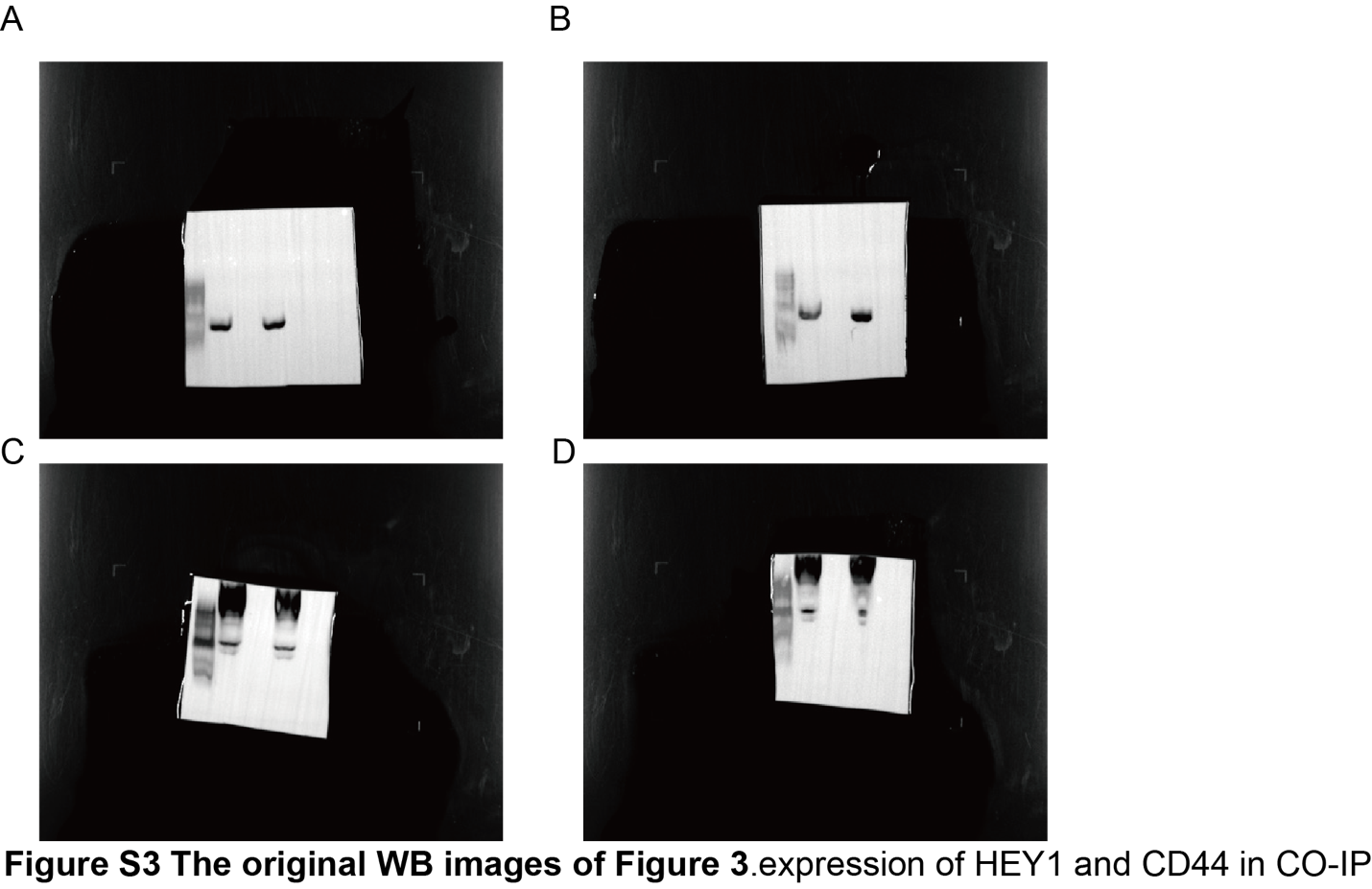


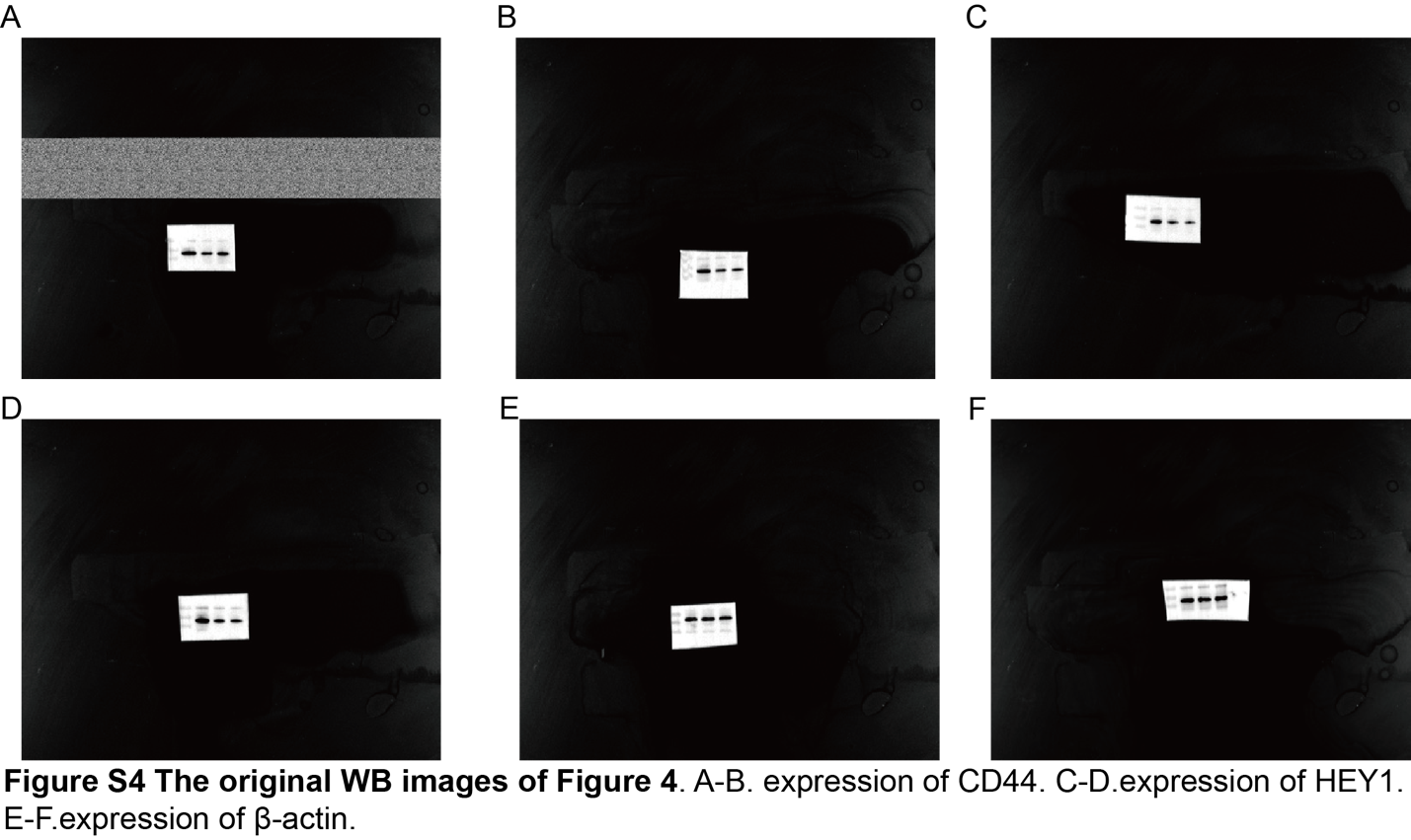


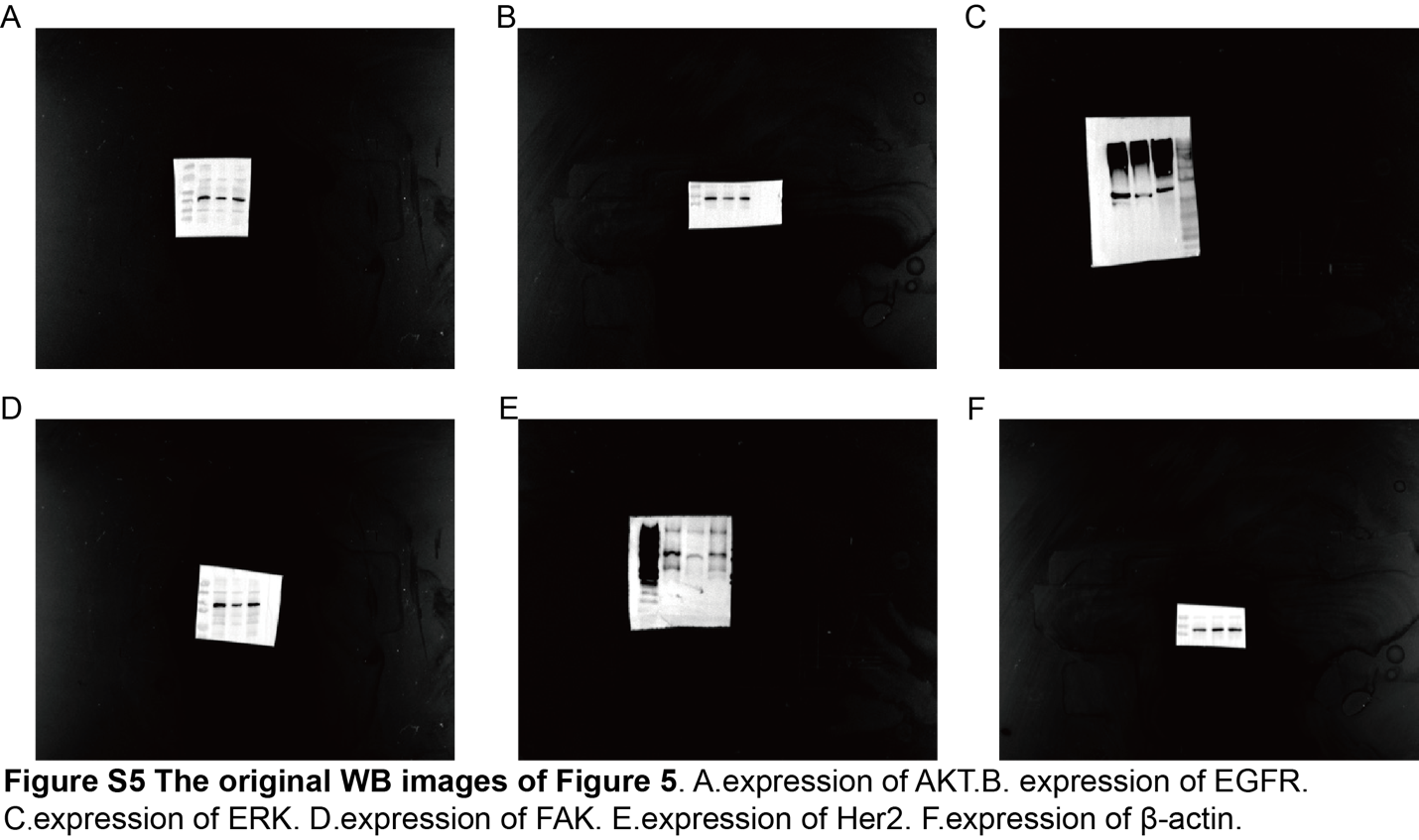

Supplement: Supplementary file 1 — Data S1. [file JCMM-29-e70042-s001.docx]
